# Screening DNA-targeted anticancer drug *in vitro* based on cancer cells DNA-templated silver nanoclusters

**DOI:** 10.1038/s41598-019-45523-2

**Published:** 2019-06-20

**Authors:** Feng Zhang, Hao Sheng, Shuang Wang, Yu Ma, Changqun Cai

**Affiliations:** 1 College of Science, Hunan Agricultural University, Changsha, 410128 China; 2 College of Resources & Environment, Hunan Agricultural University, Changsha, 410128 China; 3 Key Laboratory for Green Organic Synthesis and Application of Hunan Province, Key Laboratory of Environmentally Friendly Chemistry and Application of Ministry of Education, College of Chemistry, Xiangtan University, Xiangtan, 411105 China

**Keywords:** Fluorescent probes, Bioanalytical chemistry

## Abstract

A reliable and sensitive cancer cells DNA-templated silver nanoclusters probe has been proposed for screening DNA-targeted anticancer drugs *in vitro*. In this paper, the DNA-templated silver nanoclusters was used to investigate the binding between anthracycline antibiotics and DNA. The template of DNA-templated silver nanoclusters was extracted from human liver carcinoma cells directly, which can express the drug activities against cancer cells more direct than the normal cells DNA. The anti-tumor activities of the four drugs were validated by MTT and apoptotic assay as Mitoxantrone > Epirubicin > Daunorubicin > Adriamycin.

## Introduction

As cancer becomes a serious problem increasingly, the work of screening anti-cancer drug has gained increasing interest. Most chemotherapeutic anticancer drugs interact directly with DNA or inhibit normal DNA relaxation^1^, such as anthracycline antibiotics and other compounds. The anthracycline antitumor antibiotics have been widely used in the clinic for the treatment of a variety of cancers^2^. A great deal of biochemical evidence shows that anthracycline antibiotics mainly inhibit the process of replication and transcription by binding to DNA^3^. Usually, the binding strength between anticancer drugs and DNA is related to the biological activity of drugs^4^. Therefore, the study of drug-DNA binding can provide a reliable method for screening drugs with DNA probes. There are many reported instrument screening assays that can straightforward determine anti-drugs *in vitro*, such as UV-Vis spectroscopy^5^, nuclear magnetic resonance^6^, resonance light scattering (RLS) technique^7^ and so on^8–12^. Among them, RLS technique has received much attention because of the simplicity and sensitivity compared with others. However, the target DNA used in the reported paper was normal cells DNA. As we all known, the DNA-targeted anticancer drugs affect cancer cells DNA directly, and if the target DNA was cancer cells DNA, the result of screening method would be more reliable and intuitive. Therefore, this paper used cancer cell DNA-templated silver nanoclusters (DNA/Ag-NCs) to screen cancer drugs, which not only ensures the reliability of screening methods, but also improves the sensitivity of Ag-NCs in anticancer drugs.

Novel metal nanoclusters are a new kind of fluorescent nanomaterials and have attracted much attention of scientists due to the distinguished features of low toxicity, high fluorescent yield, good photochemical stability and small sizes^13–15^, especially Ag NCs^16–18^. In addition, silver ions possess a high affinity for DNA molecules and have the strongest binding capacity with cytosine nucleotides. Since Dickson and colleagues reported the first DNA/Ag-NCs, generous studies have been used DNA/Ag-NCs as a new fluorescent probe for monitoring different substances. Furthermore, Wang *et al*. investigated drug-DNA interaction by utilizing oligonucleotide stabilized silver nanoclusters^19^. Despite all this, there was no report about using cancer cells DNA/Ag NCs to screen anticancer drug *in vitro*.

In this paper, the activities of four anticancer drugs were screened and analyzed by DNA/Ag-NCs screening technique. The antitumor activities of these four anthracycline antibiotics were demonstrated by the RLS and fluorescence (FL) experiments directly, Mitoxantrone (MIT) > Epirubicin (EPI) > Daunorubicin (DAU) > Adriamycin (ADR), which was validated by 3-(4,5-dimethyl-2-thiazolyl) -2,5-diphen-yl-2-H-tetrazolium bromide (MTT) assay and cell apoptosis assay. The mechanism can be explained as follows primarily: the DNA-targeted anticancer drugs interact with DNA by inserting between the base pairs^20^, resulting in RLS intensity enhancing and fluorescence quenching of DNA/Ag NCs^21^. The DNA extracted from the human liver tumor cell line (SMMC-7721 cells) serves as a template for the synthesis of Ag NCs, which can more reliably screen the activity of the drug. In conclusion, a more reliable and sensitive approach was developed to screen DNA-targeted anticancer drug, which can express the drug activities against cancer cells more direct due to the participation of cancer cells DNA/Ag NCs. Furthermore, this strategy could further be applied to guide design of DNA-targeted anticancer drugs.

## Experimental

### Materials and reagents

**Figure 1:**
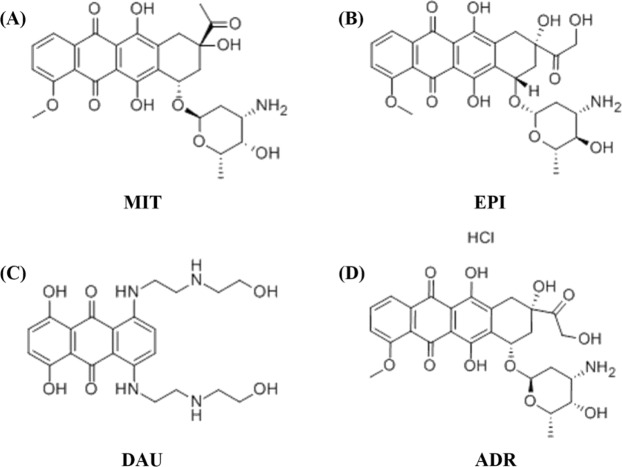
Chemical structure of the four drugs.

SMMC-7721 cells were obtained from Changsha & Information Industry Park (Liuyang National Economic & Technical Development Zone), The solutions of MIT (Rui Xin Pharmaceutical Co., Zhe Jiang, China), EPI, DAU and ADR (Hai Zheng Pharmaceutical Co., Zhe Jiang, China) were prepared in the conversation of 100.0 μg·mL^−1^. Furthermore, the structure of four drugs were shown in Fig. 1. All reagents were analytical grade and need not be further purified. Ultrapure water obtained from the Millipore water purification system (18.2 MΩ, Millipore) was used for all experiments.

### Apparatus

The RLS and FL spectra were determined using a quartz tube (1.0 cm × 1.0 cm) by a RF-5301PC fluorescence spectrophotometer (Shimadzu, Japan). The absorption spectra of the samples were determined by 100 UV-Vis spectrometer (PE, USA). The pH of the solution was measured by pHs-3C meter (Leici, Shanghai). DV-C viscometer (Brookfield Engineering Laboratories, Inc.). MR96-A microplate reader was Medical International Limited, China.

### Preparation of SMMC-7721 DNA

SMMC-7721 cells were obtained from Changsha Bio & Information Industry Park (Liuyang National Economic & Technical Development Zone) and cultured in cell culture dishes in a moist air at 37 °C with 5% CO_2_ and 95% air. The SMMC-7721 DNA was extracted with Quick Tissue/Culture Cells Genomic DNA Extraction Kit according to the manufacture’s procedures.

### Synthesis of SMMC-7721 cells DNA/Ag NCs

DNA/Ag NCs were prepared by a chemical reduction method^16^. 20 mL SMMC-7721 DNA was heated for 15 min, then cooled down quickly. 20 mM AgNO_3_ solution, 1.0 mM Sodium Citrate solution and 2.5 mM NaBH_4_ solution was added in it. Then the mixture was diluted by ultrapure water.

### RLS and FL measurements

Different concentrations of anthracycline antibiotic reserve solution and DNA/Ag-NCs were prepared in PBS. Different concentrations of drug solution were added to DNA/Ag-NCS solution. Then, the fluorescence spectra of 479–750 nm in the wavelength range were recorded, and the widths of the excitation slit and the emission slit were both 5 nm. The RLS spectra of the samples were acquired by simultaneously scanning the excitation and emission spectra (Δλ = 0 nm) in the wavelength range of 220 to 800 nm, and the slit width was 3.0 nm for excitation and emission. The enhanced RLS signal was expressed as Δ*I*_RLS_ = *I*_RLS_ − *I*_0_, where *I*_RLS_ and *I*_0_ were the RLS intensity of the DNA/Ag NCs with and without anticancer drugs, separately. The enhanced FL signal was expressed as Δ*I*_F_ = *I*_0_ − *I*_F_, where *I*_F_ and *I*_0_ were the FL intensity of the DNA/Ag NCs with and without anticancer drugs, respectively.

### MTT assay

SMMC-7721 cells were seeded in 96-well medium containing AgNCs, antibiotics, and PBS. The concentration of each antibiotic prepared in this study ranged from 0–5 μg/mL. The cells were kept for 2 h at 37 °C in a moist air of 5% CO_2_. After 72 h incubation, 20 μL MTT solution was added to each pore and kept in a humid atmosphere of 37 °C and 5% CO_2_ for 4 h. After the incubation was completed, the medium was discarded by a suction pump. Finally, 150 μL DMSO was added to each hole.

The plates were oscillated for 10 min at 37 °C and then the absorbance at 490 nm was measured using a MR96-A microplate reader. The optical density (OD) value was used to indicate the number of living cells. The 50% cell proliferation inhibitory concentration (IC_50_) of deoxynivalenol was calculated by locating the x-axis value as half of the corresponding absorbance value of the control^22^. The activity of the cells was defined as the percentage of the control group. All experiments were performed in parallel three times.

### Detection of apoptosis

SMMC-7721 liver cancer cells were cultured in a moist air of 5% CO_2_ at 37 °C for about 24 h in a 48-well culture plate inoculated with about 1 × 10^5^ cells per well. The concentration of each antibiotic prepared in this study ranged from 0.1–10 μg/mL. Apoptosis was detected by Hoechst 33342 nuclear morphology staining after induction with different concentrations of samples for 24 h. Simply, the 300 μL Hoechst 33342 staining solution was added to the pore containing 1 × 10^5^ cells and cultured at 37  °C and 5% CO_2_ for 30 min. Then, the culture solution was discarded with a suction pump and washed three times with PBS (pH 7.4). Subsequently, it was suspended in 250 mL of PBS (pH 7.4) containing 4 mg·L^−1^ Hoechst 33342 for 15 min at room temperature in the dark. Finally, under the excitation of ultraviolet light, fluorescence microscopy was used to observe and image.

## Results and Discussion

### Characterization of DNA-stabled Ag NCs

**Figure 2:**
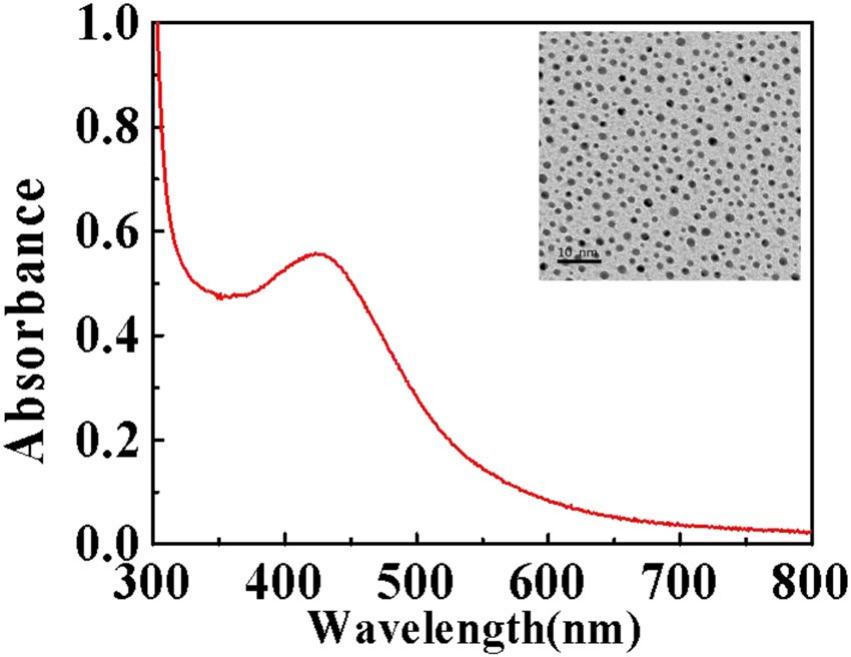
UV-Vis absorption spectrum of the DNA/AgNCs. The inserted photograph was the TEM of the DNA/AgNCs.

The synthesized DNA/Ag-NCs were characterized by UV-vis spectrophotometer and transmission electron microscopy (TEM). As shown in Fig. 2, the characteristic peaks of DNA/Ag-NCs conjugates are at 429 nm. As Ag nanoparticles have no fluorescent properties in 380–420 nm, so it was possible that DNA/Ag NCs had been formed successfully. The TEM image showed that DNA/Ag NCs have homogeneous size of about 2.0 nm (insert in Fig. 2), which can further suggest that Ag NCs were successfully prepared with DNA.

### Optical experiments

**Figure 3:**
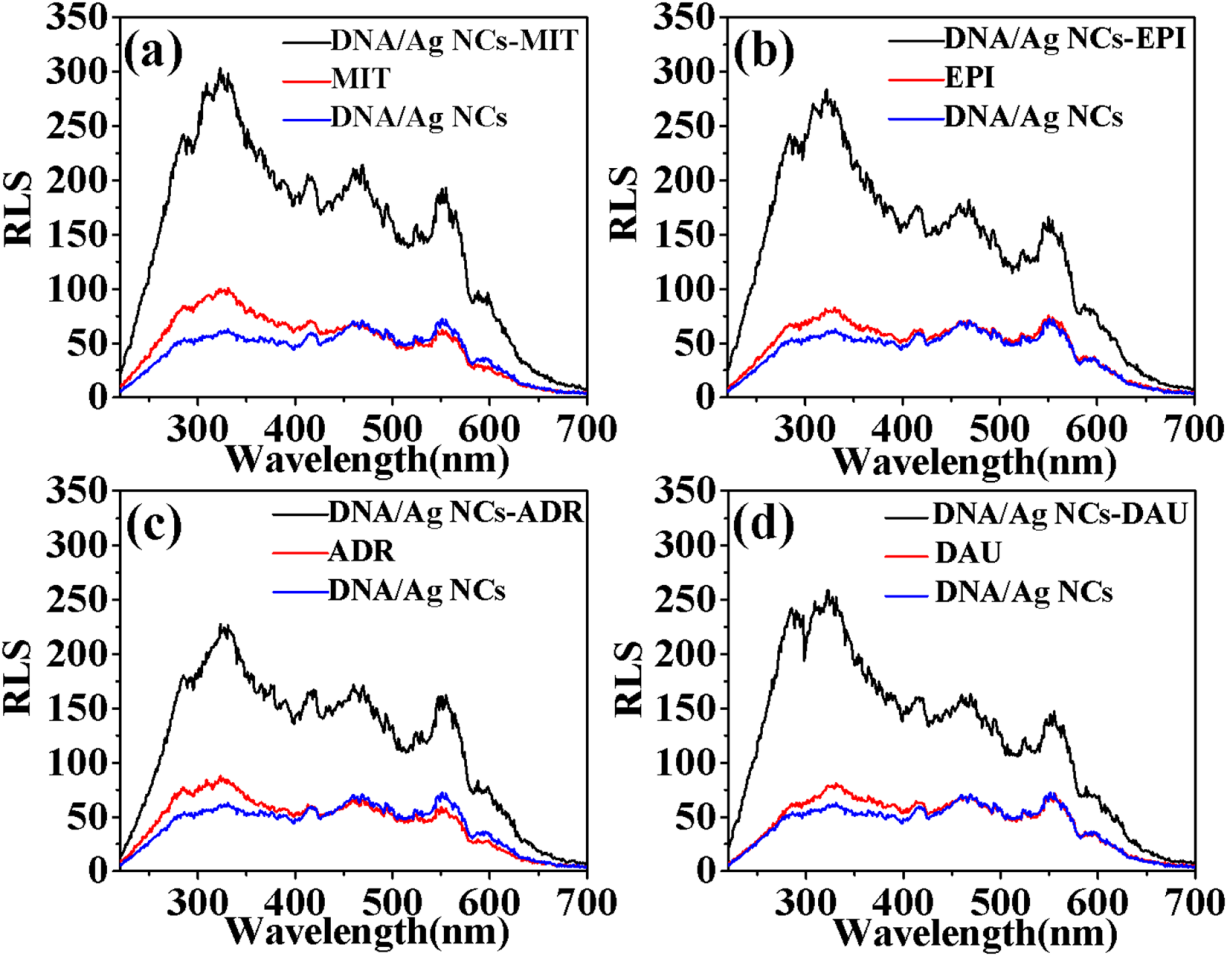
RLS Spectra of DNA/Ag NCs in combine with (**a**) MIT, (**b**) EPI, (**c**) ADR and (**d**) DAU. Experimental conditions: DNA/Ag NCs, 7.1 × 10^−5^ M; pH, 5.5.

**Figure 4:**
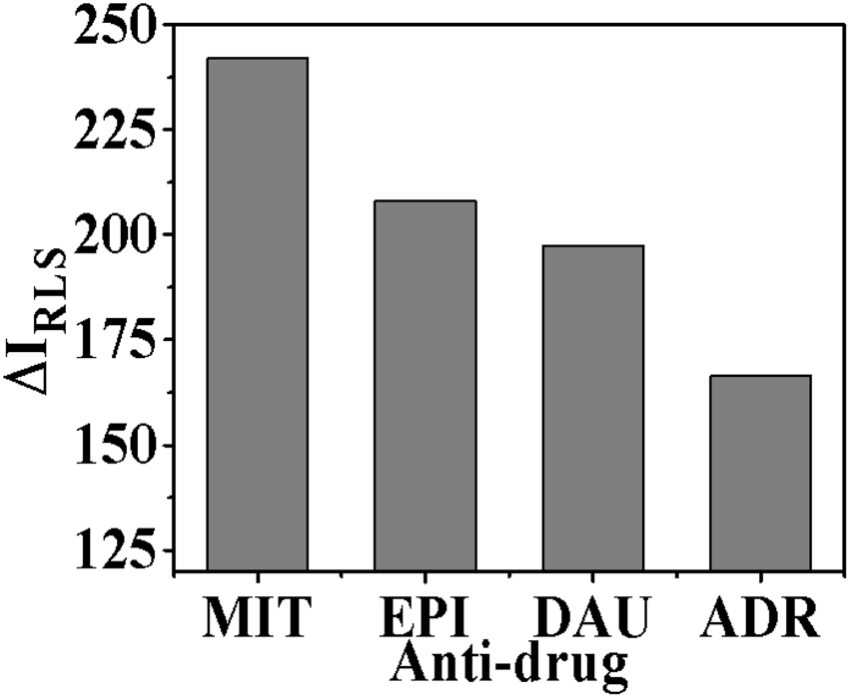
The Δ*I*_RLS_ of DNA/Ag NCs combined with MIT, EPI, ADR and DAU. Experimental conditions: DNA/Ag NCs, 7.1 × 10^−5^ M; pH, 5.5.

The shape, size and relative refractive index of the particles in the surrounding medium are the factors affecting the RLS signal. Usually, the degree of scattering will be enlarged by increasing the aggregation, resulting in greatly enhances of RLS signal. As shown in Fig. 3, it was observed that in the range of 220–800 nm the RLS signals of DNA/Ag NCs and four drugs were weak. When four drugs solution were added to solution, respectively, greatly enhanced RLS signals could be observed with RLS peaks characterized at 323 nm and 555 nm, respectively. As shown in Fig. 4, the RLS intensities had been remarkably enhanced as follows: (Δ*I*_RLS_)_MIT_ > (Δ*I*_RLS_)_EPI_ > (Δ*I*_RLS_)_DAU_ > (Δ*I*_RLS_)_ADR_. It demonstrated that DNA structure was affected by anticancer drugs. Possible explanations for this morphological effect of drug activity were considered, maybe due to intercalation effects. Usually, the binding strength between anticancer drugs and DNA is related to the biological activity of drugs, so it can be proved that the antitumor effect of these four clinical drugs is: MIT > EPI > DAU > ADR. The enhanced RLS signal exhibited an effective anticancer relationship with different anticancer drugs, and can detect concentrations as low as nanogram, indicating the establishment of a sensitive screening method for anticancer drugs.

**Figure 5:**
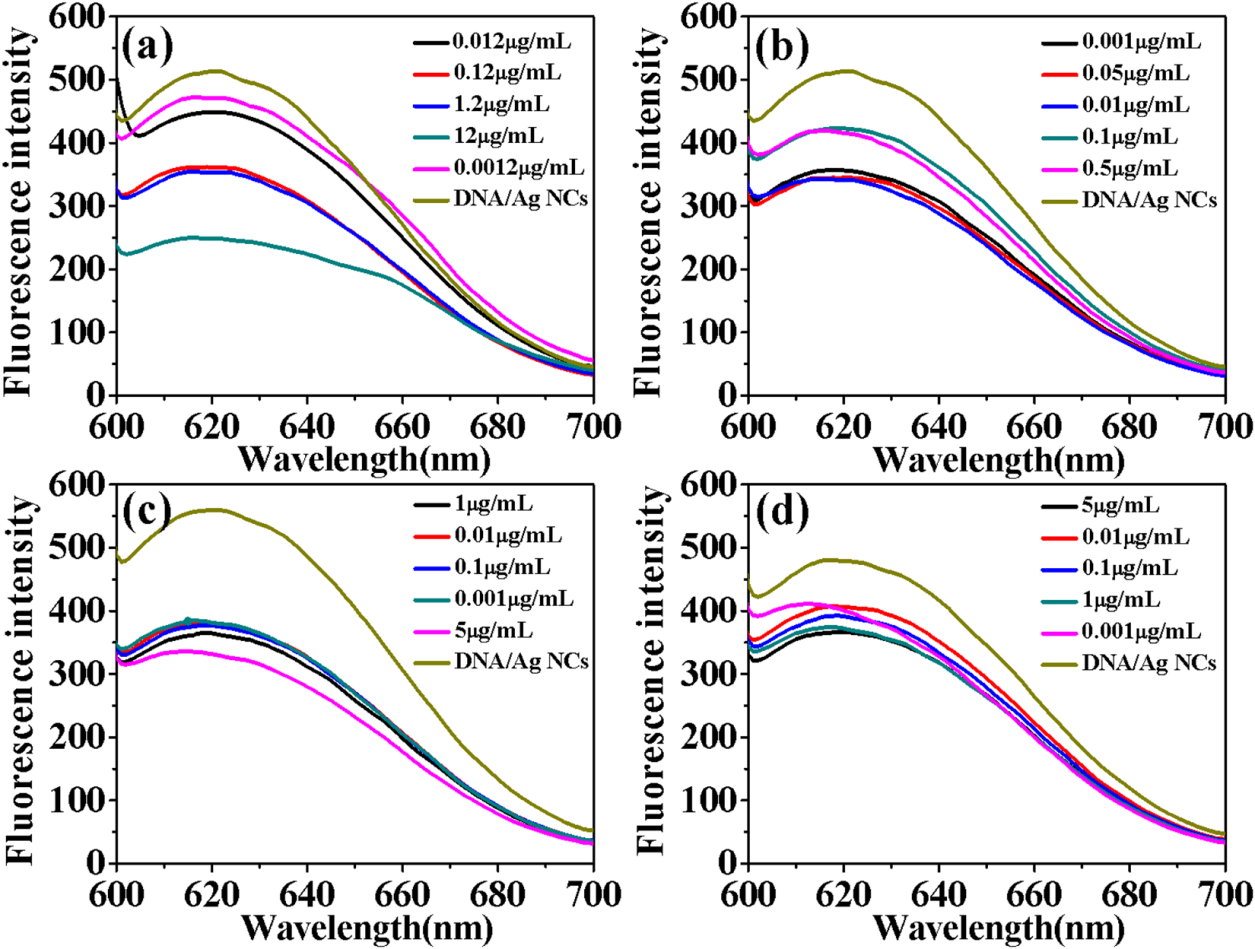
(**a**–**d**) Were the fluorescence spectra of DNA/Ag NCs under different concentration of MIT, EPI, ADR and DAU. Experimental conditions: DNA/Ag NCs, 7.1 × 10^−5^ M; pH, 5.5.

**Figure 6:**
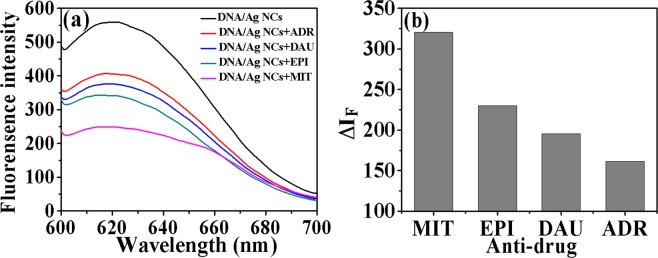
(**a**) Fluorescence spectra of different DNA/Ag NCs-drug system. Experimental conditions: Drug, 0.1 μg/mL; DNA/Ag NCs: 7.1 × 10^−5^ M; pH, 5.5; (**b**) The ΔI_F_ of DNA/Ag NCs combined with MIT, EPI, DAU and ADR. Experimental conditions: DNA/Ag NCs, 7.1 × 10^−5^ M; pH, 5.5.

The fluorescence properties of DNA/Ag NCs with a series of different concentrations of drugs were further studied. As shown in Fig. 5, when drug solution was mixed with DNA/Ag NCs solution, it could be observed that the fluorescence signal at 619 nm was greatly quenched. The DNA/Ag NCs of fluorescence signal change was related to the concentration of drug. Within a certain range, the fluorescence intensity of DNA/Ag NCs was reduced with the increasing of drug concentration. Furthermore, as shown in Fig. 6a,b, the FL intensity had been remarkably quenched as follows: (Δ*I*_F_)_MIT_ > (Δ*I*_F_)_EPI_ > (Δ*I*_F_)_DAU_ > (Δ*I*_F_)_ADR_. The fluorescence spectra of DNA/Ag NCs-drug system showed that DNA could interact with anthraquinones anti-cancer drugs, resulting in aggregation and fluorescence quenching of Ag NCs. These results also indicated the binding strength of these four clinical drugs: MIT > EPI > DAU > ADR.

**Figure 7:**
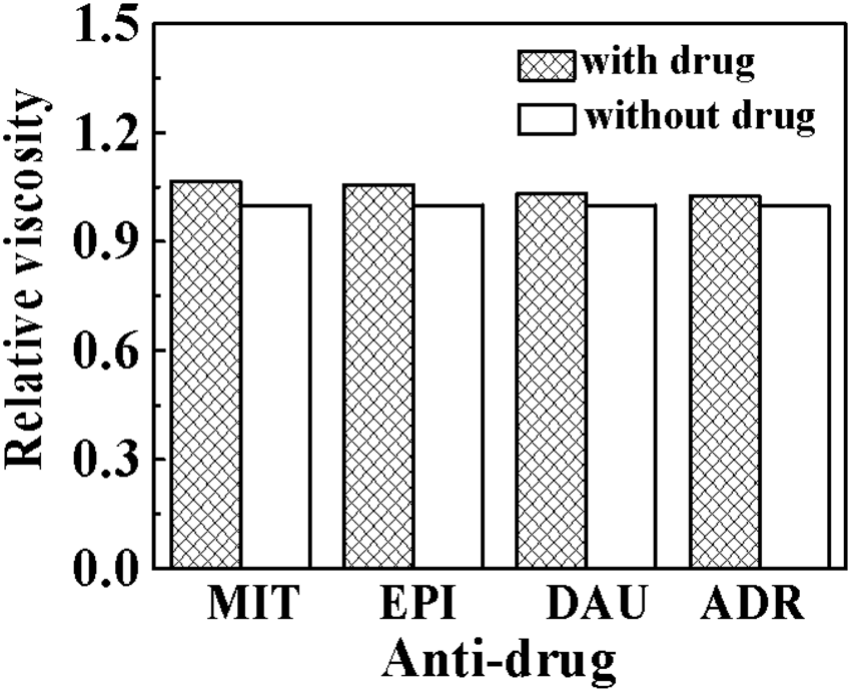
Relative viscosity with and without drug. Experimental conditions: Drug, 15.0 μg/mL; DNA/Ag NCs, 7.1 × 10^−5^ M; pH, 5.5.

In addition, the binding mode of DNA to the drug in the solution was verified by a viscosity test. Generally, there is no significant change in the viscosity of the DNA solution when the DNA interacts with the complex in a non-insertion mode such as groove surface binding or electrostatic interaction. When the complex interacts with DNA to interact in an intercalation manner, in order to allow the ligand to have enough space to enter adjacent pairs of DNA, the distance between them becomes larger, thereby allowing the DNA double helix to stretch, and the viscosity of DNA solution is enhanced. When the DNA and the complex act in a partial insertion mode, the DNA of the double helix structure may be deformed and kinked, and the viscosity of the DNA is lowered^20^. As shown in Fig. 7, when drug solution was added to DNA/Ag NCs solution, the viscosity of DNA/Ag NCs-drug system increased, indicating that the binding model of DNA and drugs is intercalation, and the Ag NCs can form a larger and more stable compound with the drug. It is also shown that (η/η_0_)_MIT_ > (η/η_0_)_EPI_ > (η/η_0_)_DAU_ > (η/η_0_)_ADR_. According to the relative viscosity, we also can get the antitumor effect of these clinical drugs: MIT > EPI > DAU > ADR.

### MTT and Apoptosis assay

**Figure 8:**
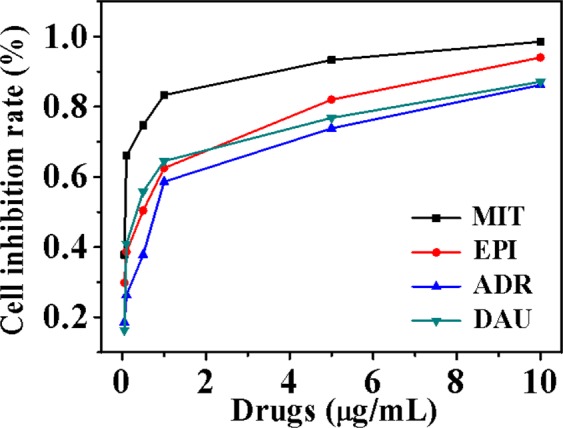
Cell inhibition rate of DNA/AgNCs-drug system. Experimental conditions: Drug, 0.01, 0.05, 0.1, 0.5, 1.0, 5.0 μg/mL; DNA/Ag NCs, 7.1 × 10^−5^ M; pH, 5.5.

As shown in Fig. 8, the cell inhibition results showed that the cell inhibition rate of DNA/Ag NCs was increased with the increase of drug concentrations within a certain range of conversation. And their antitumor effect can be obtained from the graph directly: MIT > EPI > DAU > ADR. Calculating the IC_50_ values (concentration at which 50% of the cell growth is inhibited) of SMMC-7721 cells, and the same conclusion could be got from the data of Table 1.

**Table 1:** IC_50_ of DNA-AgNCs-drug system against SMMC-7721 cells^a^.

Drugs	IC_50_
MIT	0.020
DAU	0.132
EPI	0.034
ADR	0.156

^a^IC_50_, inhibition concentration of 50% cell proliferation; SMMC-7721 cells, a human liver tumor cell line; MIT, Mitoxantrone; EPI, Epirubicin; DAU, Daunorubicin; ADR, Adriamycin.

**Figure 9:**
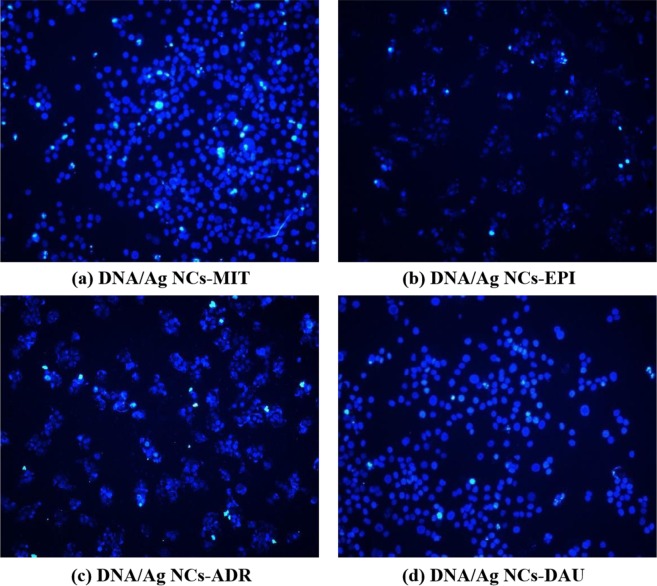
Apoptosis cells of DNA/Ag NCs-drug system. Experimental conditions: Drug, 15.0 μg/mL; DNA/Ag NCs, 7.1 × 10^−5^ M; pH, 7.4.

To better examine the mechanism of drug-induced SMMC-7721 cell death, we used Hoechst 33342 staining to examine whether the observed cell death was caused by apoptosis. When apoptosis occurs, chromatin undergoes pyknosis and apoptotic bodies appear, and the cells shrink and concentrate with fragmented chromatin^23^. Figure 9 showed the results of Hoechst 33342 staining examined with fluorescence microscopy. Cells with condensed chromatin (brightly stained) were defined as apoptotic SMMC-7721 Cells. As shown in Fig. 9, after being treated with four drugs, nuclei morphology of the cells appeared hyper condensed (brightly stained). Furthermore, the cells treated by MIT have the brightest fluorescence, indicating that dead cells are the most abundant. Followed by EPI and DAU treated cells. ADR-treated cells have the weakest fluorescence. Therefore, four drugs can promote the apoptosis of cancer cells, and the apoptosis effects of these four drugs on cancer cells are: MIT > EPI > DAU > ADR. The apoptosis rate of DNA-Ag NCs-drug system against SMMC-7721 cells was shown in Table 2, MIT has the best apoptosis effect on cancer cells, while ADR has the lowest effect. MTT and cells apoptosis experiment results are the same as the above-mentioned optical experiment results, proving that it was a reliable instrument assay in DNA-targeted anticancer drug screening.

**Table 2:** Apoptosis rate of DNA-Ag NCs-drug system against SMMC-7721 cells^β^.

Samples	Apoptosis rate
Negative control	0.05
MIT	0.113
DAU	0.068
EPI	0.088
ADR	0.057

^β^SMMC-7721 cells, a human liver tumor cell line. MIT, Mitoxantrone; EPI, Epirubicin; DAU, Daunorubicin; ADR, Adriamycin.

## Conclusion

This study developed a DNA/Ag-NCs-based probe for reliable and sensitive screening of DNA-targeted anticancer drugs, avoiding complex calculations, greatly shortening the experimental cycle and reducing the cost of the experiment. The proposed screen method not only can show that the anthraquinones anti-cancer drugs interact with DNA in the form of inserting between the base pairs, but also can indicate the activities of drugs by the RLS spectra and FL spectra directly. It is important to say that the template of DNA/Ag NCs was extracted from human liver carcinoma SMMC-7721 cells directly. In this way, it can ensure drug effects on cancer cells DNA directly and show the drug activities against cancer cells more reliable. Moreover, the screening outcomes were proved by MTT assay and apoptosis experiment, and the detection concentration of this proposed method could be as low as nanogram level, which further showed that this strategy could reliable and sensitive for DNA-targeted anticancer drug screening. In general, the proposed screening assay would be a very effective technique especially for DNA targeting drugs, and can be further used for guiding drug screening.
